# COCA-seq: genome-wide mapping of O-GlcNAc-associated open chromatin: COCA-seq maps O-GlcNAcylated open chromatin

**DOI:** 10.3724/abbs.2025207

**Published:** 2025-11-24

**Authors:** Chang Ge, Ran Zhao, Hongyu Jiang, Qingbin Chen, Zhentao Yu, Hankai Yang, Xuan Jiang, Qile Ma, Lirui Han, Kairan Yu, Guofang Li, Huang Huang, Wei Wang, Yubo Liu, Qingyue Zhang, Xing Jin

**Affiliations:** 1 College of Life and Health Sciences Northeastern University Shenyang 110000 China; 2 School of Chemical Engineering Ocean and life Sciences Dalian University of Technology Panjin 124221 China; 3 Shenzhen Salus BioMed Co. Ltd. Shenzhen 518000 China; 4 Postdoc Workstation Aim Honesty Biopharmaceutical Co. Ltd. Dalian 116000 China; 5 Department of Thoracic Surgery Cancer Hospital of Dalian University of Technology Liaoning Cancer Hospital & Institute Shenyang 110000 China; 6 Department of Anorectal Surgery The First Hospital of China Medical University Shenyang 110000 China; 7 Gastroenterology Department The First Hospital of China Medical University Shenyang 110000 China

**Keywords:** O-GlcNAcylation, chromatin accessibility, gene expression regulation, drug resistance, multiomics

## Abstract

O-GlcNAcylation, a prevalent reversible post-translational modification, intricately alters non-histone proteins, influencing the organization of gene transcriptional regulation within the accessible chromatin environment. This nucleoplasmic landscape, characterized by histone-free regions, fundamentally enables O-GlcNAc-mediated modulation through dynamic accessibility. However, unraveling the O-GlcNAc-open chromatin interplay that governs sophisticated transcriptional regulatory networks remains constrained by current techniques, which lack the resolution to probe this spatiotemporal crosstalk. Here, we report a general strategy to systematically and chemoselectively profile O-GlcNAc-associated chromatin accessibility on a genome-wide scale (COCA-seq). Through comprehensive validation across low- and high-throughput levels, we demonstrate COCA-seq’s dual fidelity in both O-GlcNAc chemoselectivity and open chromatin specificity. We employ it to delve into doxorubicin resistance for breast cancer, scrutinizing pivotal regulatory genes and transcription factors implicated in this complex biological event. By integrating bulk RNA-seq with COCA-seq, we offer a multiomics perspective, shedding light on related biological processes and pathways like drug efflux and stress homeostasis, thereby uncovering potential mechanisms by which O-GlcNAc-associated open chromatin orchestrates tumor drug resistance. COCA-seq emerges as a general and versatile tool across various biological contexts, poised to reveal the landscape of O-GlcNAc-associated open chromatin regions across the genome and decipher the significance of glycosylation behind it.

## Introduction

Chromatin, an integral component exclusive to the nucleus of eukaryotic cells, comprises a complicated assembly of nucleic acids and histones and exists in two distinct states: closed and open. Closed chromatin ensconces DNA around histones, forming nucleosomes with minimal inter-nucleosome distances, impeding both transcription and replication processes [1]. In contrast, open chromatin, characterized by its accessibility, inclining itself to transcription factors (TFs), RNA polymerase II, *etc*., thus facilitating gene expression [2]. Open chromatin regions (OCRs) represent segments of chromatin in an accessible state, which contribute to protein-DNA interactions [3]. It has been reported that OCRs contain a large number of TF binding sites and exhibit dynamic histone modifications alongside diminished DNA methylation level which is crucial for modulating gene expression, introducing phenotypic alterations and steering cellular behaviors like differentiation and development [ 1, 2, 4] .

O-GlcNAcylation, an essential post-translational modification (PTM) that attaches O-linked β-N-acetylglucosamine (O-GlcNAc) moieties to serine or threonine residues of proteins, is widespread across nuclear and cytoplasmic proteins, most notably non-histone proteins (NHPs) like TFs [5]. The critical regulatory roles of O-GlcNAc modification on chromatin-bound proteins and its influence on various facets of transcriptional regulation have been underscored in previous studies, encompassing the stability, transcriptional activity, interaction with chromatin, nuclear localization of NHPs [6]. Given the significant role of NHPs in gene expression regulation, deciphering the distribution of O-GlcNAc-modified NHPs within OCRs assumes paramount importance, offers insights into O-GlcNAc-associated chromatin accessibility and delineates lineage-specific transcriptional regulatory principles. High-throughput sequencing technologies such as DNase-seq and ATAC-seq have been extensively utilized to profile chromatin accessibility, enabling exploration of OCRs on a genome-wide scale across diverse biological processes [7]. However, there is still an absence of approaches that allow genome-wide enrichment of OCRs bound by O-GlcNAc-modified NHPs in the nucleus and accordingly investigation of their connections.

Hence, we integrated DNase-seq for chromatin accessibility profiling with O-GlcNAc metabolic glycan labeling (MGL) to develop a genome-wide strategy to chemoselectively probe O-GlcNAc-associated chromatin accessibility (Chemoselectively O-GlcNAc-Chromatin Accessibility Sequencing, COCA-seq) [8]. COCA-seq supports the exhaustive identification and enrichment of OCRs that are targeted by O-GlcNAc-modified NHPs across the entire genome, thereby facilitating the elucidation of transcriptional regulation mechanisms orchestrated by O-GlcNAc-modified NHPs within OCRs. Tumor drug resistance is not only known to be involved with genetic mutations in cancer cells, but also with a variety of non-genetic adaptive mechanisms [9]. Cancer cells transmit inherent cellular plasticity via changes in chromosome structures and functions, allowing them to rapidly adapt transcriptional and/or metabolic programs to survive drug pressure and leading to the elusiveness of such a sophisticated regulatory network [10]. Utilizing COCA-seq, we investigated doxorubicin resistance in breast cancer cells, specifically comparing MCF-7/WT (WT) and its doxorubicin-resistant counterpart MCF-7/DOX (DOX). By exploring disparities of O-GlcNAc-associated open chromatin (OGAC) between these two cell lines and dissecting differentially expressed genes (DEGs) and exclusive TFs within OCRs, we advanced the comprehensive comprehension of upstream and downstream pathways implicated in doxorubicin resistance in breast cancer. Furthermore, our findings spotlighted the significance of O-GlcNAcylation on cancer drug resistance and clinical treatment, elucidating its intricate involvement in cellular responses to chemotherapeutic agents.

## Materials and Methods

### Cell culture and reagents

The human breast adenocarcinoma cell line MCF-7 was obtained from the Type Culture Collection of the Chinese Academy of Sciences (Shanghai, China). Primary cells underwent immediate passages and snap-freezing in validated cryoprotectant solution (10% DMSO and 90% fetal bovine serum (FBS; BBI, Shanghai, China) with controlled rate. Following long-term storage in liquid nitrogen, cells were resuscitated six months pre-experiment to ensure stable growth characteristics. MCF-7 cells were cultured in the complete medium composed of 90% RPMI-1640 medium (Gibco, Carlsbad, USA) and 10% FBS, under standard conditions of 5% (v/v) CO _2_ and 37°C. Doxorubicin-resistant variant of breast cancer cells, known as DOX, was established using a previously reported drug-escalation protocol [11]. These resistant cells were maintained in RPMI-1640 medium supplemented with 10% FBS. To preserve their drug-resistant phenotype, the culture medium was supplemented with doxorubicin (Solarbio, Beijing, China) during weekly maintenance cycles, ensuring a final concentration of 1 μM. Only cell populations with a viability of over 90% after this conditioning regimen were used for experimental procedures.

### Chromatin complexes capture

Following two PBS rinses, cells were crosslinked with 1% (v/v) formaldehyde for 10 min at room temperature. The crosslinking reaction was terminated by incubating with 2.625 M glycine quenching solution for 5 min. Cells were washed and scraped from the plate, centrifuged and subjected to lysis buffer [11.25 mM Tris-HCl (pH 8.0), 11.25 mM NaCl, 45 mM KCl, and 0.05% (v/v) NP-40] with 10 min of incubation on ice to disrupt cellular membranes and alter nuclear permeability. The nuclei were subsequently washed with stabilization buffer [15 mM Tris-HCl (pH 8.0), 15 mM NaCl, 60 mM KCl, 0.15 mM spermine, 0.5 mM spermidine, 1 mM PMSF, 0.5 mM DTT, and 1× PIC (EDTA-free Protease Inhibitor Cocktail, #11836170001; Roche, Basel, Switzerland)]. Chromatin digestion was carried out by incubating the nuclei with Deoxyribonuclease I (DNase I, #2270A; TaKaRa, Dalian, China) in reaction buffer (60 mM MgCl _2_, 100 mM NaCl, 10 mM CaCl _2_, and 400 mM Tris-HCl, pH 7.9) at 37°C for exactly 2 min. The enzymatic reaction was halted by adding termination buffer (50 mM Tris-HCl, pH 8.0, 100 mM NaCl, 1% SDS, 0.3 mM spermidine, and 0.1 mM spermine) and incubating at 55°C for 15 min. The crosslinked chromatin complex fragments were then obtained [12].

### Metabolic labelling and click reaction

Cells at 30% confluence were cultured in 10-cm dishes with complete medium containing 1,6-di-O-propionylated N-azido acetylgalactosamine (1,6-Pr _2_GalNAz, kindly provided by Prof. Bo Cheng of Peking University, Beijing, China) for 48 h [13]. The crosslinked chromatin of metabolically labeled cells was then captured as described above. Meanwhile, a small portion of the reaction mixture was set aside as input. For the selective biotinylation of O-GlcNAz-modified proteins, the chromatin was incubated with rotation in a solution containing 100 μM alkyne-biotin (#1266-25; Click Chemistry Tools, Scottsdale, USA), 250 μM BTTAA (#1236-100; Click Chemistry Tools), 500 μM CuSO _4_, and 2.5 mM freshly prepared sodium ascorbate for 2 h at room temperature, protected from light. Subsequently, an eightfold volume of methanol was added to the mixture, which was then stored at ‒80°C overnight. The precipitated proteins were collected by centrifugation at 4000 *g* for 15 min at 4°C and resuspended in PBS. After washing the streptavidin magnetic beads (#22305-10; BEAVER Life Science, Guangzhou, China) three times with PBS, the beads were transferred with the above solution. The resulting mixture was incubated on a rotating rack for 3‒5 h at room temperature. To minimize nonspecific binding, the beads were washed with PBS containing 1% SDS.

### Immunoblotting assay

For immunoblotting of O-GlcNAz-tagged proteins, samples were quantified using the Bicinchoninic Acid (BCA) protein quantification assay kit (#P0012; Beyotime, Shanghai, China). The collected beads were mixed with SDS-PAGE loading buffer and boiled for 10 min. The denatured samples were separated on 10% SDS-polyacrylamide gels under constant voltage. The gels were either stained with Coomassie Brilliant Blue R-250 or processed using a silver staining kit (#P0017S; Beyotime) for total protein visualization. When immunoblotting, the resolved proteins were transferred onto polyvinylidene fluoride (PVDF) membranes (Immobilon-P; Millipore-Sigma, Billerica, USA) at a constant current. The membranes were blocked with 5% (w/v) bovine serum albumin (BSA) in PBST [PBS with 0.05% (v/v) Tween-20] for 4 h at room temperature. Biotinylated proteins were detected by incubating with streptavidin-conjugated horseradish peroxidase (#3999; Cell Signaling Technology, Beverly, USA) diluted 1:20,000 in PBST for 45 min at room temperature followed by three washes with PBST. Chemiluminescent signals were developed using an ECL Plus kit (#SQ202; Epizyme, Shanghai, China) and captured digitally using the ChemiDoc MP Imaging System (Bio-Rad, Hercules, USA). All immunoblots shown represent data from at least two independent experiments.

### Nucleic acid agarose gel electrophoresis

Agarose gel (1%) was prepared with agarose and TAE buffer, with ethidium bromide (EB) added for visualization. DNA samples were mixed with loading buffer and loaded into sample wells. Electrophoresis was performed at a constant voltage suitable for DNA sizes. After electrophoresis, gels were visualized under UV light using a gel documentation system, and the results were photographed. The migration of the DNA samples was compared with DNA ladders (TaKaRa) to estimate the molecular weight of the DNA fragments. Experiments were independently repeated at least twice.

### COCA-seq and bioinformatics

Approximately 2.5 × 10 ^6^ cells per immunoprecipitation (IP, pulled down by streptavidin) were used for COCA-seq analysis. Magnetic beads were prepared as described above. The beads were resuspended in PBS (containing 1% SDS) and digested with RNase A (#2158; TaKaRa) at 37°C for 1 h, followed by three washes. Proteinase K (#3115828001; Roche) was then added and incubated for 9 h at 55°C. DNA was purified using the MiniBEST DNA Fragment Purification Kit Ver.4.0 (#9761; TaKaRa). The optimal DNA fragment size is mainly concentrated between 50 and 600 bp to exhibit clear periodic peaks, especially those centered around 150‒200 bp, along with distinct nucleosome-free region peaks. These fragment sizes are critical for revealing chromatin accessibility and nucleosome structure. Fragments longer than 800 bp typically indicate low tagmentation efficiency, which may compromise data quality. DNA fragments (100‒750 bp) within OCRs were isolated by 1% agarose gel electrophoresis using the MiniBEST Agarose Gel DNA Extraction Kit Ver.4.0 (#9762; TaKaRa). The purified DNA was subjected to library preparation, including end repair, A-tailing, adapter ligation, and amplification. The prepared DNA library was used for next-generation sequencing (NGS) on an Illumina NovaSeq 6000 instrument (Novogene Technology, Gibbstown, USA) and/or Salus Pro gene sequencer (Shenzhen Salus BioMed Co., Ltd., Shenzhen, China), with two biological replicates sequenced. The raw COCA-seq, COGC-seq, and ATAC-seq data were aligned to the hg19 human reference genome using BWA (v0.7.18) and samtools (v1.20) [14]. Enriched binding peaks were identified using MACS2 (v2.2.9.1) [15]. MACS2 calculates the *p* value for each candidate peak using an exact Poisson test based on a dynamic local background λ estimated from multiple genomic windows to capture local biases. To control for multiple testing, *p* values were adjusted using the Benjamini-Hochberg procedure, and peaks passing the significance threshold were reported. The counts of mapped reads in peak regions were normalized using DiffBind (v3.12.0), which includes edgeR (v4.0.16), and differential quantification regions were determined (|fold change| ≥ 1.5 and FDR ≤ 0.05) [16]. Bigwig files were generated using deepTools (v3.5.4) and visualized in the Integrative Genomics Viewer (IGV; v2.16.2). The genomic distribution, annotation comparison, and visualization of OGAC were analyzed using bedtools (v2.31.1) and ChIPseeker (v1.38.0) [17]. Motif discovery and enrichment analysis were performed using peak sequences based on the RGT-HINT (HMM-based Identification of TF footprints; v1.0.2) [18]. Sequencing signal density heatmaps and sample Pearson correlation coefficients were generated by deepTools. GO functional enrichment analysis was conducted using Metascape, and GSEA was performed using the R package clusterProfiler (v4.10.0) [ 19‒ 21] .

### RNA sequencing

RNA-seq assays were conducted as previously described [22]. In brief, RNA was extracted from 5 × 10 ^4^ WT or DOX cells. The RNA was then fragmented and reverse transcribed to generate a cDNA library. Sequencing of the library was performed using an Illumina NovaSeq 6000 instrument (Novogene Technology) and/or Salus Pro gene sequencer (Shenzhen Salus BioMed Co., Ltd.). Two biological replicates were included in the RNA-seq analysis. Trim-galore (v0.6.4) was utilized to remove reads with more than 10% unknown bases and low-quality reads. STAR (v2.7.11a) was employed to align the clean reads to the reference genome (hg19) [23]. Subsequently, featureCounts (v2.0.3) was used to quantify the RNA-seq reads mapped to genomic features in the BAM files. Low-abundance transcripts were filtered out by retaining only genes with an average count greater than 1 across all samples to reduce noise and improve the accuracy of downstream analysis. DEGs were identified using DESeq2 (v1.42.1), with a fold change threshold of 1.5 and a *p* adj of ≤ 0.05 [24]. Additional bioinformatics analyses were carried out using various packages and functions in R software, including ggplot2 and tidyverse.

### TCGA data analysis

Transcriptomic data and corresponding clinical annotations for 664 breast invasive carcinoma (BRCA) patients were retrieved from The Cancer Genome Atlas (TCGA) database. The patient cohort was divided into two groups: one consisting of 349 patients who received anthracycline-based chemotherapy (with doxorubicin as the chemotherapeutic agent), and the other comprising 315 treatment-naïve controls. Overall survival (OS) analysis was conducted using the R statistical environment (v4.3.2). Kaplan-Meier survival curves were generated with the survival package (v3.8.3) via the survfit function. The survminer package (v0.5.0) was employed to visualize the survival curves. Potential confounding clinical factors, such as tumor subtype, stage, and hormone receptor status, were considered unlikely to substantially affect the OS analysis and therefore were not included as covariates. Log-rank tests were used to compare survival differences between the two groups, with a significance level set at *p*  ≤ 0.05.

### qPCR

Total RNA was extracted using the TRIzol reagent (Invitrogen, Carlsbad, USA) and then reverse transcribed and amplified with the PrimeScript™ RT Master Mix (Perfect Real Time, #RR036A; TaKaRa) and PCR Thermal Cycler Dice™ Gradient (TaKaRa) following the manufacturer’s instructions. Quantitative real-time PCR (qPCR) for DNA was performed using TB Green® Premix Ex Taq™ II (Tli RNaseH Plus) (#RR820A; TaKaRa) and a LightCycler® 96 Instrument (Roche). For qPCR of COCA-seq samples, DNA within OGAC was obtained as described above, then decrosslinked and purified for qPCR. Amplification was carried out with primers specific to the indicated open regions of target genes. Primers were designed using Primer-BLAST (NCBI) and Oligo 7, with amplicon lengths limited to 80‒200 bp. WT and DOX cells without metabolic labeling served as negative controls. All primer sequences used in this study are provided in Supplementary Tables S1 and S2.

### Quantification and statistical analysis

All statistical analyses were conducted using GraphPad Prism 10.1.2 and Microsoft Excel 2021. This study was also supported by the Galaxy platform as appropriate [25]. Statistical significance was defined as *p* value ≤ 0.05. Prior to analysis, the variation within each data group and the assumptions of the tests were thoroughly examined. Data are presented as the mean ± SEM, and the similarity of data variances was verified. Two-sided unpaired Student’s *t*-test was used to assess statistical differences in Gaussian-distributed data. To ensure reproducibility, blots or photographs were repeated at least twice, as indicated in the specific methods and figure legends. COCA-seq and RNA-seq was performed with two biological replicates. Other experiments were repeated three times, with each independent replicate including at least three technical replicates. All results were successfully reproduced. Source data and original uncropped blots with molecular weight markers are archived in Supplementary Materials 1 and 2. The sequencing data generated in this study have been deposited in GEO database (GSE294152 and GSE294153). Publicly available datasets analyzed include: WT and DOX cells ATAC-seq (GSE174152) [26], COGC-seq (GSE141698) [11], WT cells ChIP-seq for H3K4me3 and H3K27ac (GSE97481) [27], H3K9ac (GSE95898) [28], and H3K4me1 (GSE86714) [28]. The corresponding authors will provide the original data used to support the findings of this study upon reasonable request. Source data are provided with this paper.

## Results

### COCA-seq is chemically designed and developed with the combination of a nuclease and click chemistry

We initiated our study by determining approaches to analyze chromatin accessibility. TFs and other NHPs that directly bind to OCRs are critical regulators in mediating gene replication and transcriptional activation [ 1, 29] . Notably, DNA segments devoid of histone are more vulnerable to fracture or disruption by nucleases, transposases, or physical and chemical treatment, resulting in fragments of varying lengths. For that reason, open chromatin is obtained mostly by cutting up the DNA in OCRs with enzymatic digestion or sonication. DNase I is an enzyme which non-selectively cleaves single- or double-stranded DNA [30]. When chromatin is treated with low concentrations of DNase I, fracture occurs at specific sites known as DNase I hypersensitive sites (DHSs), these DHSs are typically found in OCRs without nucleosomes [31]. When integrating DNase I with NGS, DHSs can be efficiently and specifically identified across the genome, enabling the detection of OCRs. DNase-seq has become the “gold standard” for assessing chromatin accessibility and can also determine NHP binding sites within OCRs at single-base resolution, providing insights into the occupancy of TFs and other NHPs [30]. Therefore, in our study, DNase-seq was selected as one of the core methods to design and develop COCA-seq, allowing us to investigate chromatin accessibility comprehensively.

To selectively enrich O-GlcNAc-modified NHPs, we adopted the MGL strategy as our second core method. MGL strategy combines metabolic incorporation of clickable non-natural monosaccharides with click chemistry for functional probe attachment, which can exclusively target and enrich O-GlcNAc-modified proteins, thereby unraveling O-GlcNAc-associated chromatin accessibility [8]. This strategy offers versatility as various common non-natural monosaccharides can undergo interconversion through metabolic pathways, achieving the labeling of diverse glycoproteins. For O-GlcNAc labeling we utilized N-azido acetylgalactosamine (GalNAz), an azido analog of N-acetylgalactosamine (GalNAc), which is ultimately converted into UDP-N-azido acetylgalactosamine (UDP-GalNAz) within the cell and then interconverts with UDP-N-azido acetylglucosamine (UDP-GlcNAz) via the glycometabolic pathway [32]. The resulting UDP-GlcNAz serves as the substrate and forms O-GlcNAz tags via O-glycosidic bonds on serine or threonine residues of proteins. Since partially protected non-natural monosaccharides can easily cross the cell membrane to attain efficient labeling in glycometabolism without introducing side reactions, we used 1,6-Pr _2_GalNAz in our MGL strategy.

**Figure 1:**
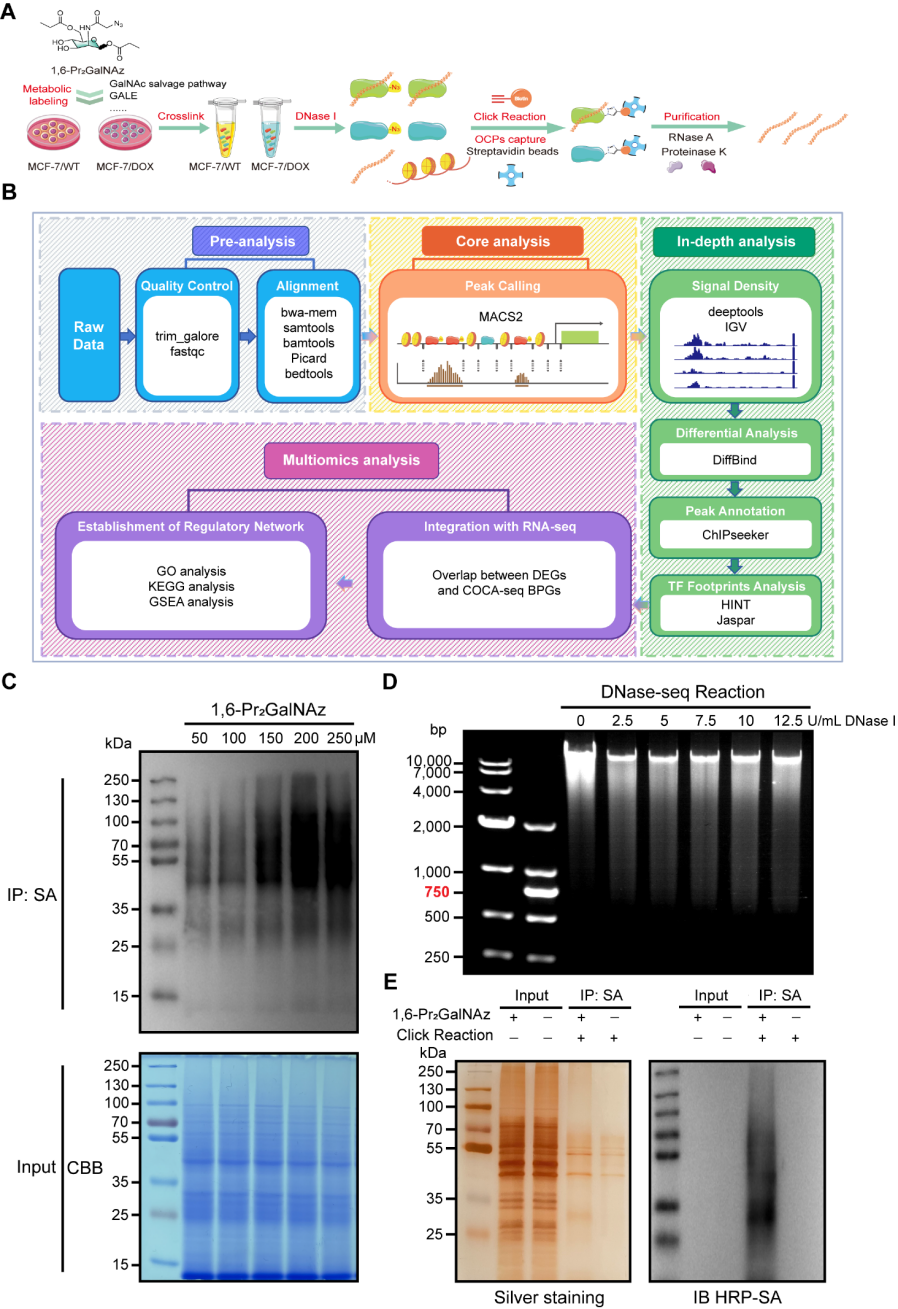
Figure 1 Establishment and optimization of the COCA-seq strategy (A) Schematic overview of the chemoselective O-GlcNAc-chromatin accessibility sequencing. Both WT and DOX cells were cultured with 1,6-Pr2GalNAz. Following metabolic labeling, genomic DNA fragments covalently bound to O-GlcNAc-modified NHPs were treated by DNase I, isolated via decrosslinking and processed for high-throughput sequencing. (B) Bioinformatics analysis pipeline for COCA-seq data. Raw COCA-seq reads underwent rigorous quality control before alignment to the human reference genome (hg19) using the BWA algorithm. Genomic regions exhibiting significant enrichment signals were identified through peak calling with MACS2. Differential peak analysis was performed using DiffBind to categorize peaks into two groups: DOX-biased peaks (enriched in DOX cells) and WT-biased peaks (enriched in WT cells). To elucidate the biological relevance of these differential peaks, peak-associated genes and TF binding motifs were systematically analyzed. In parallel, RNA-seq data were processed with DESeq2 to identify DEGs between WT and DOX cells. Finally, integrative analysis of COCA-seq and RNA-seq datasets was conducted to delineate the O-GlcNAc-regulated transcriptional network underlying doxorubicin resistance. (C) Immunoblotting of metabolically labelled WT cell lysates. Total cellular proteins were labeled with 1,6-Pr2GalNAz (50–250 μM, 48 h), conjugated to alkyne-biotin, and probed with streptavidin to assess the best metabolic labelling efficiency. 200 μM 1,6-Pr2GalNAz was identified as the optimal concentration for subsequent O-GlcNAc studies. (D) Agarose gel electrophoresis of formaldehyde-crosslinked chromatin fragments following DNase I digestion (0–12.5 U/mL, 2 min) and subsequent treatment with RNase A and Proteinase K. 5 U/mL DNase I was determined for generating chromatin fragments enriched in accessible genomic regions. (E) Immunoblotting and silver staining of WT Input and IP chromatin complexes after DNase I digestion. For (C–E), all blots or photographs were representative of at least two biologically independent experiments.

In our study, breast cancer cells WT and its doxorubicin-resistant strain DOX were cultured with 1,6-Pr _2_GalNAz, and subsequently, NHPs were cross-linked to chromatin in 1% formaldehyde. This formaldehyde-mediated cross-linking captured interactions between O-GlcNAc-modified NHPs and open chromatin. Chromatin fragmented by low concentrations of DNase I further underwent the copper(I)-catalyzed azide alkyne cycloaddition (CuAAC), while the alkyne had been attached to biotin [33]. Then streptavidin beads were utilized to enrich OGAC, followed by treatment with RNase A and proteinase K, and later sequencing ( Figure 1A). Ultimately, sequencing yielded sufficient reads which would go through a workflow of bioinformatics analysis ( Figure 1B) [34]. To tailor COCA-seq for breast cancer cells, we implemented the most suitable experimental parameters. Specifically, we optimized the culture conditions for 1,6-Pr _2_GalNAz by omitting the crosslinking step and extracting whole O-GlcNAc-modified proteins for subsequent SDS-PAGE analysis. This optimization ensured efficient metabolic labeling ( Figure 1C). Furthermore, we systematically determined the ideal DNase I treatment conditions by quantitatively analyzing DNA fragmentation patterns using agarose gel electrophoresis, which allowed for precise and reproducible digestion of chromatin ( Figure 1D). Importantly, COCA-seq strategy successfully and precisely captured O-GlcNAc-modified NHPs, as evidenced by immunoblotting and silver staining of formaldehyde-crosslinked chromatin complexes ( Figure 1E).

### COCA-seq establishes chemoselective profiling of O-GlcNAc-associated chromatin accessibility

**Figure 2:**
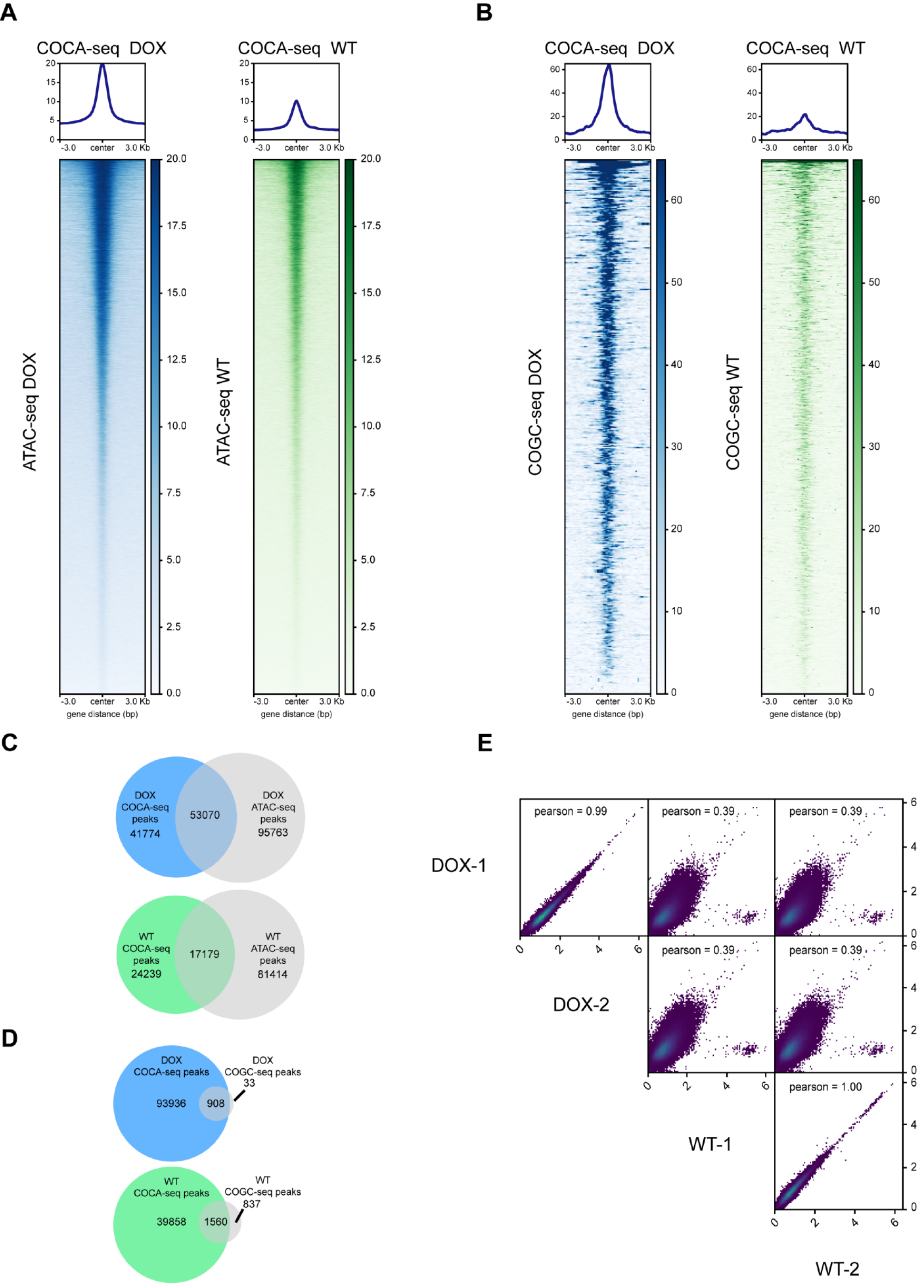
Figure 2 COCA-seq specifically captures OGAC validated through bioinformatics (A,B) Average read density profiles (top) and corresponding heatmaps (bottom) of COCA-seq signals at genomic regions defined by ATAC-seq and COGC-seq peaks. Enrichment levels (white: low; blue/green: high) were analyzed across a ±3 kb window centered on peak summits. (A) Comparative analysis with ATAC-seq data (profiling for accessible chromatin) (GSE174152). (B) Comparative analysis with COGC-seq data (O-GlcNAcylated chromatin enrichment data) (GSE141698). (C,D) Venn diagrams depicting the intersection between COCA-seq peaks and chromatin regions identified by ATAC-seq or COGC-seq. (C) Overlap with ATAC-seq-defined accessible regions. (D) Overlap with COGC-seq-identified O-GlcNAc-enriched regions. For (A–D), biological duplicates are also shown in Supplementary Figure S1. (E) Correlation analysis of COCA-seq BigWig signal intensities demonstrating intra-group reproducibility and inter-group heterogeneity. Pearson correlation coefficients are shown as numerical values.

Based on the dual specificity from the chemoselectivity of the MGL strategy and the precision in targeting DHS by DNase I, we hypothesized that DNA fragments enriched through COCA-seq would predominantly originate from OGAC. To validate this hypothesis, we compared COCA-seq data with published ATAC-seq and COGC-seq datasets. High-resolution profiling revealed broad WT and DOX signal distributions with characteristic unimodal peaks at both ATAC-seq and COGC-seq peak centers, demonstrating conserved chromatin features ( Figure 2A,B and Supplementary Figure S1A,B). Notably, COCA-seq peaks exhibited substantial genomic overlap with ATAC-seq peaks, confirming its capacity to resolve conventional OCRs ( Figure 2C and Supplementary Figure S1C). Significant concordance between COCA-seq and COGC-seq peaks was observed, highlighting the unique selectivity of COCA-seq for O-GlcNAc-modified chromatin regions ( Figure 2D and Supplementary Figure S1D). Collectively, these results underscored the inherent chemoselectivity for O-GlcNAc modification of COCA-seq and its spatial precision in OCR detection. Additionally, COCA-seq demonstrated high reproducibility between biological replicates, with pronounced differences between groups ( Figure 2E).

### COCA-seq delineates distinctions in O-GlcNAc-associated chromatin accessibility landscapes of drug-resistant breast cancer

**Figure 3:**
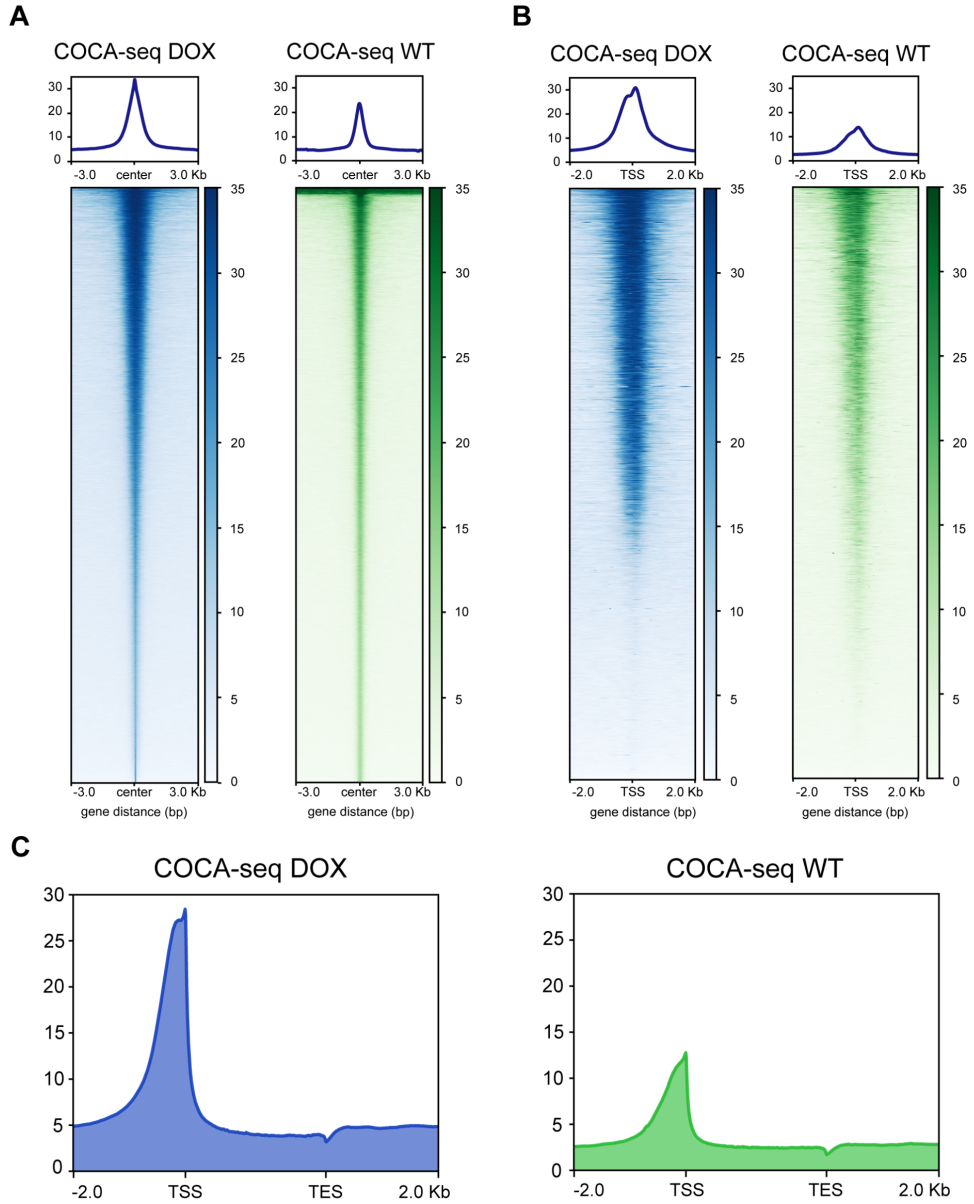
Figure 3 COCA-seq signals significantly enrich at promoters in doxorubicin-resistant breast cancer cells implied elevated transcriptional activity (A,B) Heatmap visualization and average signal profiles of COCA-seq read density. (A) Signal distribution centered on COCA-seq peak summits (±3.0 kb). (B) Signal distribution flanking TSSs (±2.0 kb). The blue/green color indicates a high signal. (C) Genome-wide average enrichment profiles of COCA-seq signals in DOX and WT cells. Profiles spanned annotated gene regions (from TSS to TES (transcription end site)) based on hg19 reference genome. For (A–C), biological duplicates are also shown in Supplementary Figure S2.

Drawing from our previous research, the elevated gene expression activity observed in DOX implied the emerging drug resistance [11]. To substantiate the differences in OGAC between DOX and WT, we examined the genome-wide distribution of COCA-seq signals. Noticeable divergence in chromatin accessibility patterns was observed, with DOX exhibiting epigenetic signatures distinct from WT. Specifically, we scrutinized the distribution of COCA-seq signals at transcription start sites (TSSs) and peak centers. While both DOX and WT displayed sharp single peaks, DOX presented strikingly higher summits at these regions consistently ( Figure 3A–C and Supplementary Figure S2A–C). This indicated that DOX possessed higher overall chromatin accessibility, which likely contributed to its increased transcriptional activity and cellular function, thereby supporting global chromatin remodeling associated with drug resistance. Moreover, minimal correlation between DOX and WT also statistically validated distinctive chromatin features underlying the drug-resistant phenotype ( Figure 2E).

### COCA-seq characterizes cell-type-specific OGAC architectures with different functional profiles

**Figure 4:**
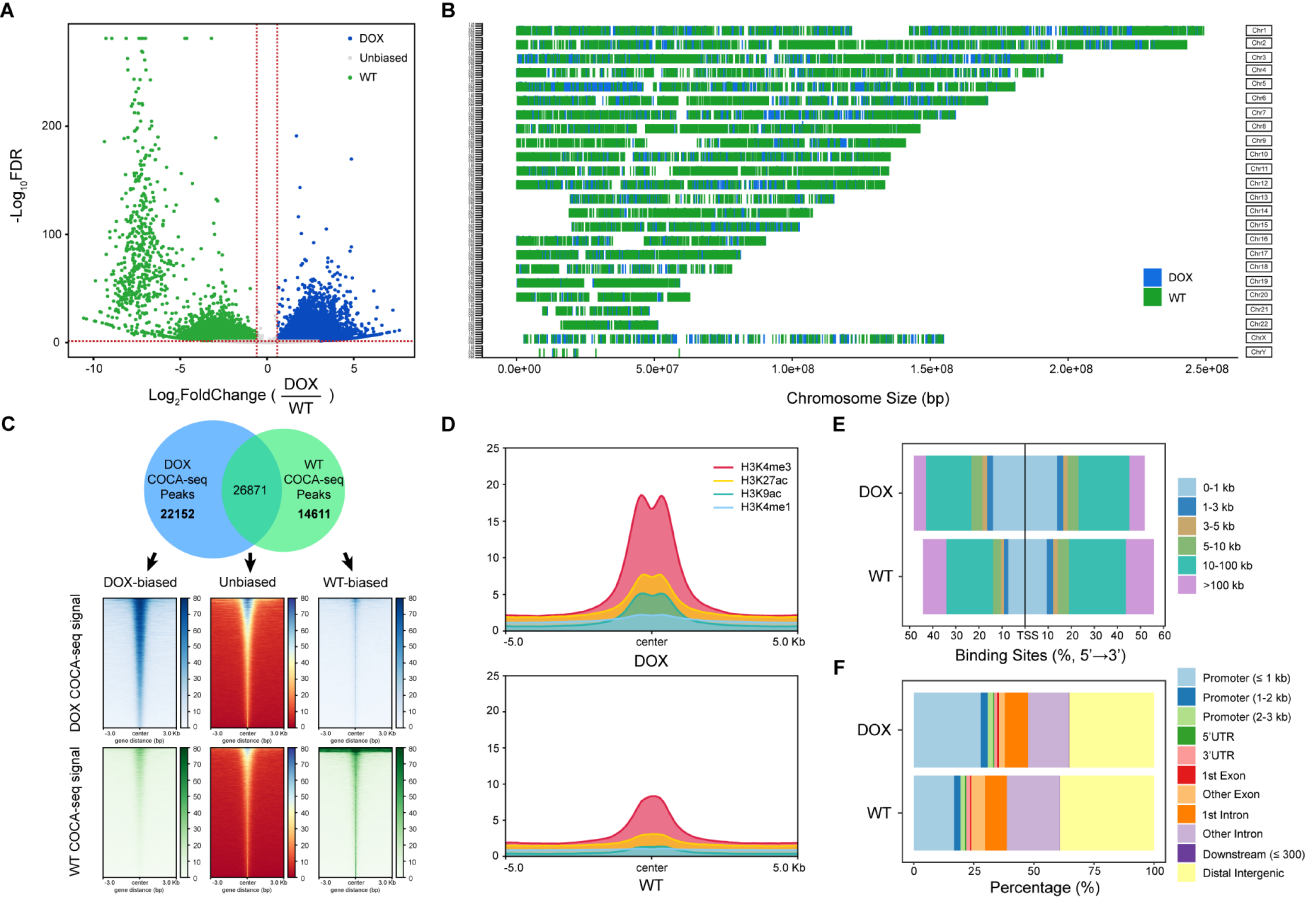
Figure 4 COCA-seq identifies transcriptional activation and oncogenic reprogramming in doxorubicin-resistant breast cancer through O-GlcNAc-associated chromatin accessibility (A) Scatter plot of DiffBind-identified biased peaks (|fold change| ≥ 1.5, FDR ≤ 0.05) based on binding affinity scores. Differential binding analysis was performed using edgeR, which models count data with a negative binomial distribution and p values were adjusted for multiple testing using the Benjamini-Hochberg procedure to control FDR. (B) Chromosomal distribution of DOX-biased (blue) and WT-biased (green) peaks across the reference genome. (C) COCA-seq occupancy signals of identified WT-biased peaks, DOX-biased peaks and unbiased peaks. Top: Venn diagram illustrating the overlap of COCA-seq peaks between DOX and WT. Bottom: COCA-seq signal enrichment heatmaps (±3.0 kb from peak centers; white/red: low; blue/green: high). (D) Average enrichment of published WT ChIP-seq reads for four histone modifications H3K4me3, H3K27ac (GSE97481), H3K9ac (GSE95898) and H3K4me1 (GSE86714) within ±5.0 kb of biased COCA-seq peak centers. (E) Genomic annotation of biased OGAC by ChIPseeker, categorized into exons, intergenic regions, introns, 3′UTR, 5′UTR, and promoters. (F) Distribution of distances from biased accessible regions to the nearest TSS.

Through peak calling, a total of 118,168 and 94,844 peaks were identified for DOX, and 47,212 and 41,418 peaks for WT. To elucidate the role of these OGAC regions (namely COCA-seq peaks) in doxorubicin resistance, we utilized DiffBind to filter out differentially accessible regions, which resulted in 22,152 DOX-biased peaks and 14,611 WT-biased peaks (|fold change| ≥ 1.5 and FDR ≤ 0.05) ( Figure 4A). The chromosomal distribution of these biased peaks was markedly different between DOX and WT ( Figure 4B,C). We further deciphered biased COCA-seq peaks using ChIPseeker. It was found that there were 10,217 O-GlcNAc-related genes in OGAC of DOX, including many previously unreported O-GlcNAc-targeted genes, as well as 7438 genes in WT.

Chromatin accessibility, nucleosome positioning, and three-dimensional chromosome organization are intricately regulated by histone modifications, which modulate the interaction between transcriptional complexes and DNA, thus dictating local genomic properties and transcriptional states [ 1, 35] . Investigation of histone modifications from the genome or specific regions can provide insights into gene activation status, the positioning of regulatory elements like promoters and enhancers, and their functions in disease pathogenesis, particularly in cancer and immunological disorders. Histone methylation marks such as H3K4me1 (typically found at enhancers) and H3K4me3 (found at promoters) are tied to boosted gene expression [ 35, 36] . Similarly, histone acetylation marks such as H3K27ac and H3K9ac, at enhancers and promoters, signifies chromatin structure relaxation and gene activation [37]. Analysis of publicly available ChIP-seq data revealed increased activation-associated histone modification signals around the centers of DOX-biased COCA-seq peaks, confirming enhanced transcriptional competence in DOX compared to WT ( Figure 4D). Furthermore, the identified biased OGAC covered a variety of functional regions across the genome, such as promoters, 5′UTRs, 3′UTRs, protein-coding exons, introns, and distal intergenic regions. Surprisingly, DOX-biased OGAC regions were more enriched in promoters than those of WT, especially within 1 kb of TSSs, which suggested the establishment of an active chromatin state conducive to transcriptional complex assembly ( Figure 4E,F). Overall, these findings highlighted the superior transcriptional activity in DOX, which may ultimately position O-GlcNAc-associated chromatin accessibility as an epigenetic rheostat controlling drug resistance transcriptional programs.

### COCA-seq dissects vital biological processes and regulatory pathways manipulating drug resistance in breast cancer through illustration for differential expression of genes in cell-biased OGAC

**Figure 5:**
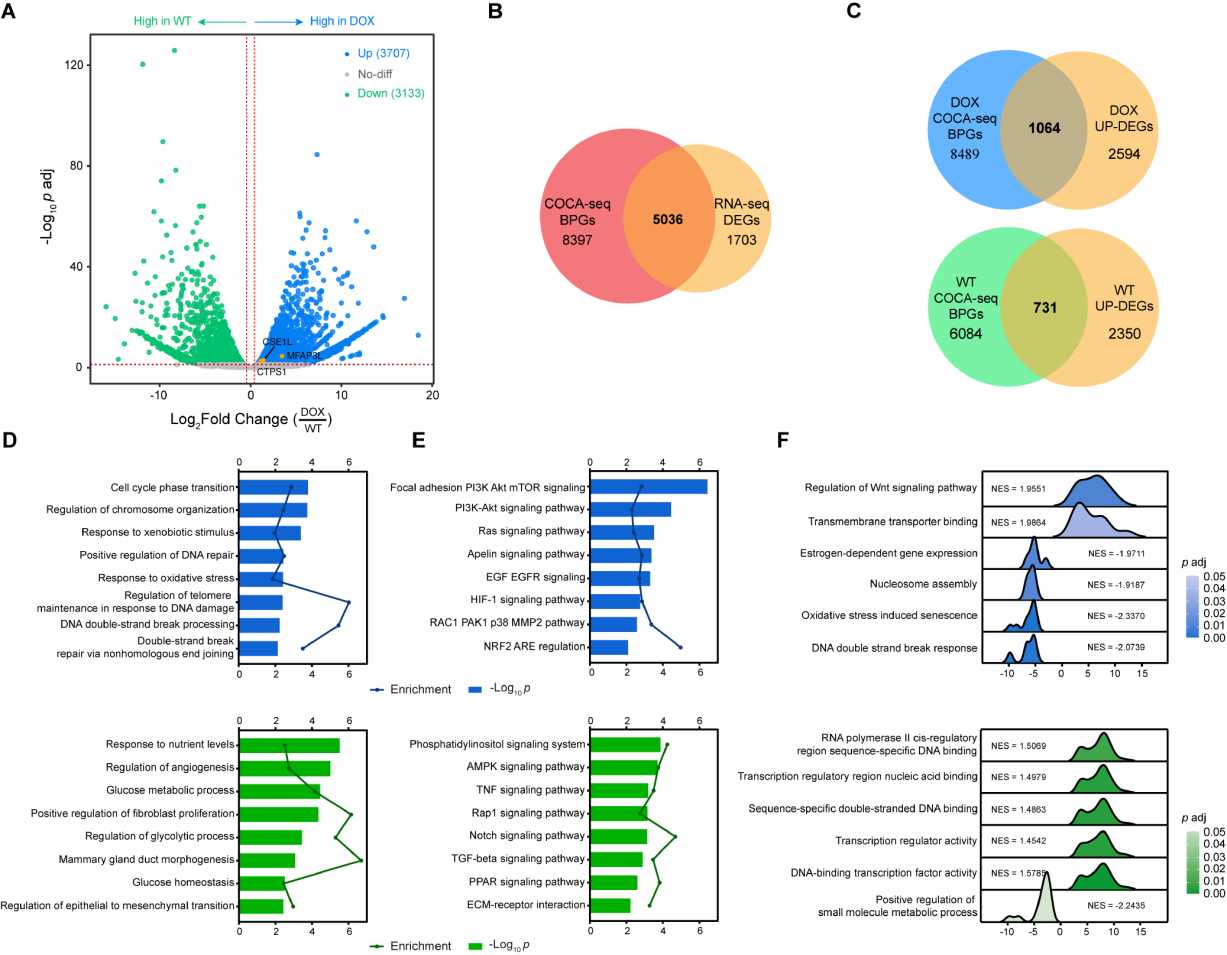
Figure 5 COCA-seq coupled with transcriptomics reveals regulatory networks driving chemoresistance in breast cancer (A) Volcano plot of RNA-seq differential expression analysis. A total of 3707 genes were significantly upregulated in DOX cells (blue), and 3133 genes were upregulated in WT cells (green) (|fold change| ≥ 1.5, p adj ≤ 0.05). The adjusted p values were obtained from Wald test and Benjamini-Hochberg procedure. The genes mentioned in this study later are labeled. (B) Venn diagram illustrating the overlap between DEGs from RNA-seq and COCA-seq BPGs. (C) Venn diagrams showing the overlap of DOX- and WT-highly expressed DEGs with corresponding COCA-seq BPGs. (D,E) Functional enrichment analysis of upregulated BPDGs (1064 in DOX and 731 in WT) identified in (C). (D) GO analysis highlighting important biological processes. (E) KEGG pathway analysis revealing vital regulatory pathways. Enrichment scores and p values are labeled. (F) Ridgeline plots of GSEA results for BPDGs in DOX and WT cells. Normalized enrichment scores (NES) and p adj values (from permutation-based nominal p values and Benjamini-Hochberg procedure) are indicated. Darker colors correspond to higher statistical significance.

To elucidate transcriptional alterations of COCA-seq biased peak-associated genes (BPGs) between DOX and WT, we integrated RNA-seq with COCA-seq, aiming to comprehensively assess BPGs expression on a genome-wide scale. Gene expression profiling by RNA-seq revealed 6840 DEGs ( *p* adj ≤ 0.05 and |fold change| ≥ 1.5), with 3707 genes exhibiting elevated activity in DOX and 3133 in WT ( Figure 5A). By conducting an integrative multiomics analysis, we compared RNA-seq DEGs (6739 genes) with COCA-seq BPGs (13,433 genes) and yielded 5036 biased peak-associated DEGs (BPDGs), further characterizing the BPDGs highly expressed in each cell type ( Figure 5B,C). Genes that are both BPGs and DEGs were defined as the BPDGs, representing the overlapping subset in the multi-omics analysis. Overall, this multiomics approach significantly improved the resolution of drug resistance-associated transcriptional networks compared to conventional single-omics analyses, demonstrating the potential of COCA-seq as a powerful tool to improve the precision of clinical decision-making.

KEGG analysis of BPGs suggested that the pathways enriched in DOX were associated with drug resistance ( Supplementary Table S3). The heightened expression of DOX-BPDGs likely brought about modulated cellular activities and in turn doxorubicin resistance. Focusing on the highly expressed BPDGs (BPHGs), we constructed the regulatory networks they participated in, providing deeper insights into the impact of O-GlcNAc modification on doxorubicin resistance in breast cancer. GO analysis showed that DOX-BPHGs were predominantly enriched in biological processes related to stress response, intracellular homeostasis, and DNA damage repair. Conversely, WT-BPHGs were more associated with malignant tumor development, including metastasis, invasion, and solid tumor progression ( Figure 5D). Regarding pathway remodeling induced by OGAC, KEGG analysis highlighted the prominence of PI3K-Akt signaling pathway, NRF2 ARE regulation and RAC1 PAK1 p38 MMP2 pathway in DOX, while Notch signaling pathway, TGF-β signaling pathway, and ECM-receptor interaction were more pronounced in WT ( Figure 5E). To quantitatively reveal the significant differences in biological states between DOX and WT, we performed GSEA analysis on COCA-seq BPDGs, uncovering preferentially activation of drug efflux and stress homeostasis, and downregulation of stress response, apoptosis and senescence in DOX, contrasting with processes associated with TF binding and gene expression modulation were enhanced in WT ( Figure 5F). Taken together, doxorubicin resistance in breast cancer may stem from coordinated suppression of drug-induced stress response and shifting priorities for such exogenous stimuli, such as DNA damage repair, drug efflux, and cell survival, rather than malignancy progression or even tumor metastasis.

It is evident that COCA-seq possesses a remarkable capacity to elucidate the intricate regulatory mechanisms mediated by O-GlcNAc. As demonstrated in the study, COCA-seq explicated that acquired doxorubicin resistance involved metabolic-stress adaptation through O-GlcNAcylation-mediated chromatin remodeling, effectively decoupling cancer cells from progression-associated transcriptional programs. COCA-seq exhibited unprecedented resolution in dissecting O-GlcNAc-driven regulatory complexity, particularly in mapping chromatin accessibility.

### COCA-seq maps the cell-specific TF profiles regulated by O-GlcNAc

**Figure 6:**
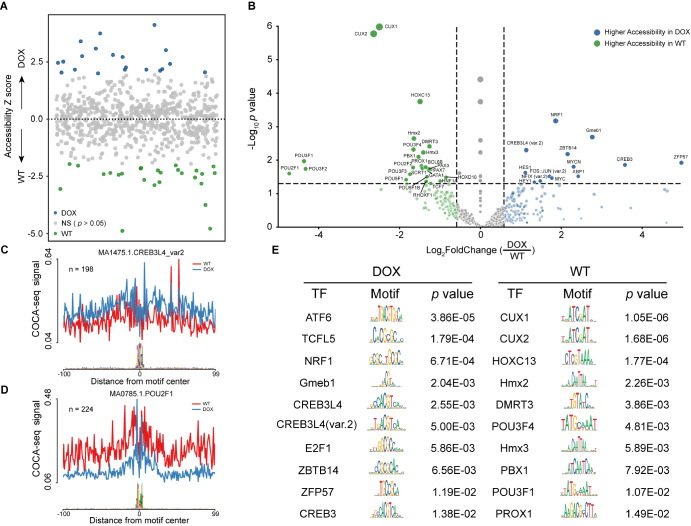
Figure 6 COCA-seq delineates O-GlcNAc-mediated TF binding dynamics through genome-wide analysis of biased chromatin accessibility motifs (A) Scatterplot of TF binding activity in DOX and WT biased peaks derived from HINT analysis. This plot illustrated dynamic changes in TF activity between DOX and WT cells. (B) Volcano plot of TF activity fold changes between DOX and WT cells. TFs with significant changes in activity scores (|fold change|≥ 1.5, p adj ≤ 0.05) are labeled. (C,D) Distribution probability of two TFs’ binding motifs within COCA-seq biased peak regions. Higher COCA-seq signal intensity reflected increased TF activity in the corresponding cell type. (C) CREB3L4_var2 in DOX. (D) POU2F1 in WT. Gmeb1, FOS::JUN_var2, POU3F1, and GATA1 are shown in Supplementary Figure S3. (E) The top 10 significant cell-specific TFs.

Given the substantial enrichment of OGAC in the promoter regions of DOX, we hypothesized that the TF profiles interacting with these regions might critically regulate drug resistance. To support this hypothesis, we utilized HINT, a computational framework that leveraged open chromatin data to detect active TF binding sites [18]. DOX and WT displayed distinct TF activity patterns ( Figure 6A,E). Further analysis identified 38 differentially active TFs (|fold change| ≥ 1.5, FDR ≤ 0.05) between the two groups, with 13 TFs in DOX and 25 in WT, including known mediators of O-GlcNAc-associated drug resistance ( Figure 6B).

Notably, NRF1, previously reported to be involved in O-GlcNAc-mediated doxorubicin resistance in breast cancer, was among the TFs with elevated activity in DOX [11]. Mechanistically, genotoxic stress induced by doxorubicin triggers O-GlcNAc modification of NRF1, activating its regulatory role in DNA damage repair and cellular stress alleviation. Similarly, among those TFs, XBP1 as a key regulator of endoplasmic reticulum stress responses, may facilitate cellular adaptation to protein misfolding and oxidative damage under doxorubicin challenge [ 37, 38] . Additionally, MYCN probably modulates doxorubicin resistance through transcriptional control of ATP-binding cassette transporters [ 39, 40] . These findings collectively implicated O-GlcNAc-modified TFs as pivotal regulators of doxorubicin resistance.

Consistent with our hypothesis, binding probability analysis revealed specific TFs with high binding activity around the centers of DOX-biased peaks, including Gmeb1, FOS::JUN_var2, CREB3L4_var2, and so on ( Figure 6C and Supplementary Figure S3A). Conversely, TFs such as GATA1, POU3F1, and POU2F1 exhibited higher binding frequencies near WT-biased peaks ( Figure 6D and Supplementary Figure S3B). COCA-seq underscored the O-GlcNAc-associated divergence in TF regulatory profiles, delineating their context-specific roles in drug-resistant and wild-type cellular states.

### COCA-seq reveals O-GlcNAc-associated potential prognostic indicators and therapeutic targets

**Figure 7:**
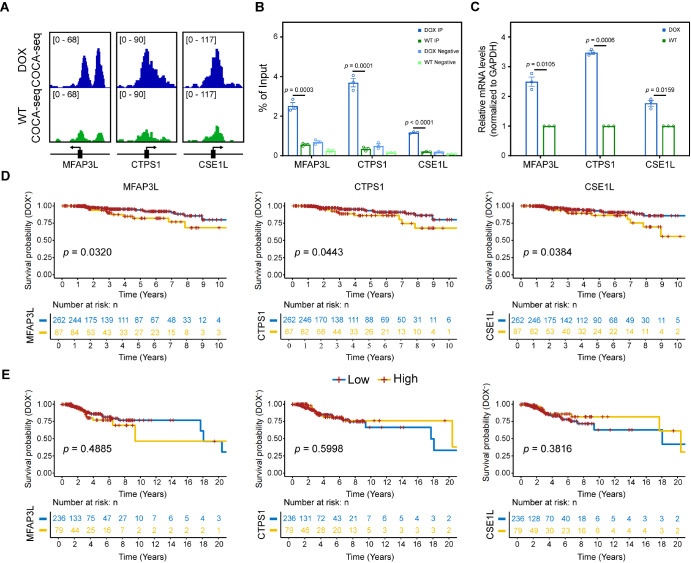
Figure 7 COCA-seq and clinical data expose prognostic relevance of O-GlcNAc-associated BPDGs in breast cancer doxorubicin treatment (A) IGV tracks of COCA-seq BigWig signals at the promoter regions of MFAP3L,CTPS1, and CSE1L. (B) qPCR analysis of MFAP3L,CTPS1, and CSE1L mRNA levels in DOX and WT cells. Expression levels were normalized to that of GAPDH. (C) Validation of chromatin accessibility by COCA-qPCR. Enrichment percentages shown are relative to input. DOX and WT cells without metabolic labeling served as negative controls. For (B,C), data are expressed as the mean ± standard error of the mean (SEM). n = 3 biologically independent experiments. p values were generated by two-sided unpaired Student’s t-tests after data had been tested for normal distribution and homogeneity of variance test. (D,E) Kaplan-Meier curves for OS analysis of BRCA patients grouped according to MFAP3L,CTPS1, and CSE1L mRNA expression in tumor tissue. Patients with gene expression levels TPM above the upper quartile are indicated by the yellow line (high group), and those below the upper quartile are indicated by the blue line (low group). Statistical analyses were performed using the log-rank test, and p values are indicated. (D) Dox+ patients (n = 349). (E) Dox– patients (n = 315).

To further validate the expression pattern and clinical significance of BPDGs in DOX and to predict their roles in the development of acquired resistance to doxorubicin in breast cancer patients, we selected several DOX-BPHGs to assess. *MFAP3L*, *CTPS1*, and *CSE1L* showed markedly higher signal intensity in the promoters of *DOX* than WT, with more pronounced peaks ( Figure 7A). Correspondingly, subsequent qPCR confirmed that these three genes were enriched for OGAC in the promoters, and their expression levels were higher in the drug-resistant strain ( Figure 7B,C), suggesting that O-GlcNAc served as a critical regulator in chemoresistance modulation. Then we retrieved expression and clinical data from TCGA-BRCA. We analyzed the expression levels of the three DOX-BPHGs and their effects on the responses of breast cancer patients treated with doxorubicin alone (Dox ^+^; *n* = 349) and those untreated (Dox ^–^; *n* = 315). Interestingly, patients with higher mRNA levels of *MFAP3L*, *CTPS1*, and *CSE1L* exhibited shorter OS times and disappointing consequences in the Dox ^+^ group ( *p* ≤ 0.05; Figure 7D). Notably, this prognostic correlation was absent in Dox ^-^ patients ( Figure 7E), indicating treatment-specific clinical significance. These results highlighted that the O-GlcNAc-mediated regulation of BPHGs, such as *MFAP3L*, *CTPS1*, and *CSE1L*, played a key role in the development of doxorubicin resistance in breast cancer. The genes identified by COCA-seq might serve as O-GlcNAc-associated important targets and prognostic biomarkers for determining the prognosis of doxorubicin-treated breast cancer patients.

## Discussion

O-GlcNAcylation, a crucial PTM, extensively modifies NHPs such as TFs [6]. O-GlcNAc-modified NHPs affect transcriptional regulation and alter gene expression levels by binding to OCRs [11], regions of chromatin that are in a state of accessibility and essential for DNA-protein interactions. Therefore, it is pivotal to explore the contribution of OGAC to gene regulatory networks. Although several high-throughput sequencing technologies are available for studying OCRs, there is a pressing need for methods integrating multiomics approaches to map OGAC comprehensively. To address this need, we developed COCA-seq by combining the MGL strategy with DNase-seq. COCA-seq successfully enabled chemoselective probing of O-GlcNAc-associated chromatin accessibility, offering a genome-wide perspective on the open chromatin interacting with O-GlcNAc-modified NHPs. Additionally, O-GlcNAc labeling via the MGL strategy ensures excellent chemoselectivity, excluding other intracellular glycosylation [13].

We applied COCA-seq to investigate regulatory mechanisms underlying drug resistance in breast cancer and illuminated that O-GlcNAc modification, as nutrient-responsive PTM, orchestrated adaptive phenotypic shifts in metabolic-stress adaptation by dynamically regulating metabolic enzymatic activity and signaling cascades. In detail, we discriminated differential OGAC in DOX and WT, predicting potential TFs and providing a valuable gene dataset to decipher the crosstalk between glycosylation and chromatin accessibility. The TFs inferred by COCA-seq may serve as key mediators through which O-GlcNAc contributes to doxorubicin resistance in breast cancer, thereby broadening the perspective for further investigation of the regulators influencing this complex cellular state. While COCA-seq provides a potent prediction of potential TFs, integrating proteomics in future studies would offer complementary and highly reliable validation, greatly enhancing the confidence and depth of the findings. Coupling COCA-seq with RNA-seq allowed us to systematically analyze BPDGs and discover related biological processes and critical regulatory pathways which induced doxorubicin resistance. Due to the high coverage of COCA-seq across the whole genome, we also identified some previously unreported biological processes. Our findings underscored the vital influence of O-GlcNAcylation on gene expression, particularly in stress homeostasis and DNA damage repair pathways, impacting breast cancer drug resistance and providing insights for future therapeutic interventions.

The potential of COCA-seq extends beyond breast cancer researches, promising broad in multiomics studies across various diseases besides cancers. COCA-seq is also capable of revealing prognostic markers and potential treatment targets. Importantly, COCA-seq paves the way for further exploration at the single-cell level into the glycosylation-based gene regulatory networks reconstruction within open chromatin and the resulting cell-type specificity. The combined application of COCA-seq and RNA-seq is mutually reinforcing and sheds light on the development of quantitative analysis of O-GlcNAc-associated gene expression differences tuned by accessible chromatin. Incorporating proteomics is likely to be more helpful to reveal functions of O-GlcNAc-modified NHPs, which bind to OCRs in a variety of physiological and pathological processes. In addition, other unnatural monosaccharides, serving as metabolic substitutes for GalNAc, can be employed for tagging and detecting glycoproteins within cells, and collectively enhance genome-wide coverage. In addition, labeling other important glycosylation such as O-fucosylation is an interesting future direction [ 41, 42] .

This study has made significant progress, yet some technical limitations persist. The MGL using 1,6-Pr _2_GalNAz effectively prevents side reactions and shows great cell membrane permeability and metabolic labeling efficiency. However, it lacks subcellular spatial specificity and cannot distinguish between GalNAz and GlcNAz labeling. In this study, we overcame this limitation and isolated nuclear GlcNAz-labeled NHPs through nuclear fractionation. For different cell types, it is necessary to systematically optimize metabolic labeling concentrations and DNase I treatment conditions. Currently, this technique is still in its early stages. It cannot resolve single-cell heterogeneity and has insufficient temporal resolution to capture rapid dynamics caused by transient stimuli, limiting its application to steady-state cell populations. Despite these challenges, this study has established the first methodological framework for investigating the crosstalk between O-GlcNAc modification and chromatin accessibility, offering a new technical advance for epigenetic regulation research. Future studies will focus on developing subcellular localization labeling probes, creating single-cell detection strategies, and improving temporal resolution to track dynamic events. These advancements will greatly expand the applicability and depth of this technique.

## Supporting information

Supplementary_Material_2

469Supplementary_Material_1

Supplementary_information_figures_and_tables.docx

## Supplementary Data

Supplementary data are available at *Acta Biochimica et Biophysica Sinica* online.
