# Supplementary material for: COCA-seq: genome-wide mapping of O-GlcNAc-associated open chromatin: COCA-seq maps O-GlcNAcylated open chromatin
Source: Acta Biochim Biophys Sin (Shanghai). 2025 Nov 24;58(5):975–88. doi: 10.3724/abbs.2025207 (PMC13214506; doi:10.3724/abbs.2025207)
Supplement: Supplementary_Material_2 [file Supplementary_Material_2.docx]

***Supplementary Material***

This file includes the full uncropped Gels and Blots images of **Figure 1C-1E**.

**Figure 1C:**

**
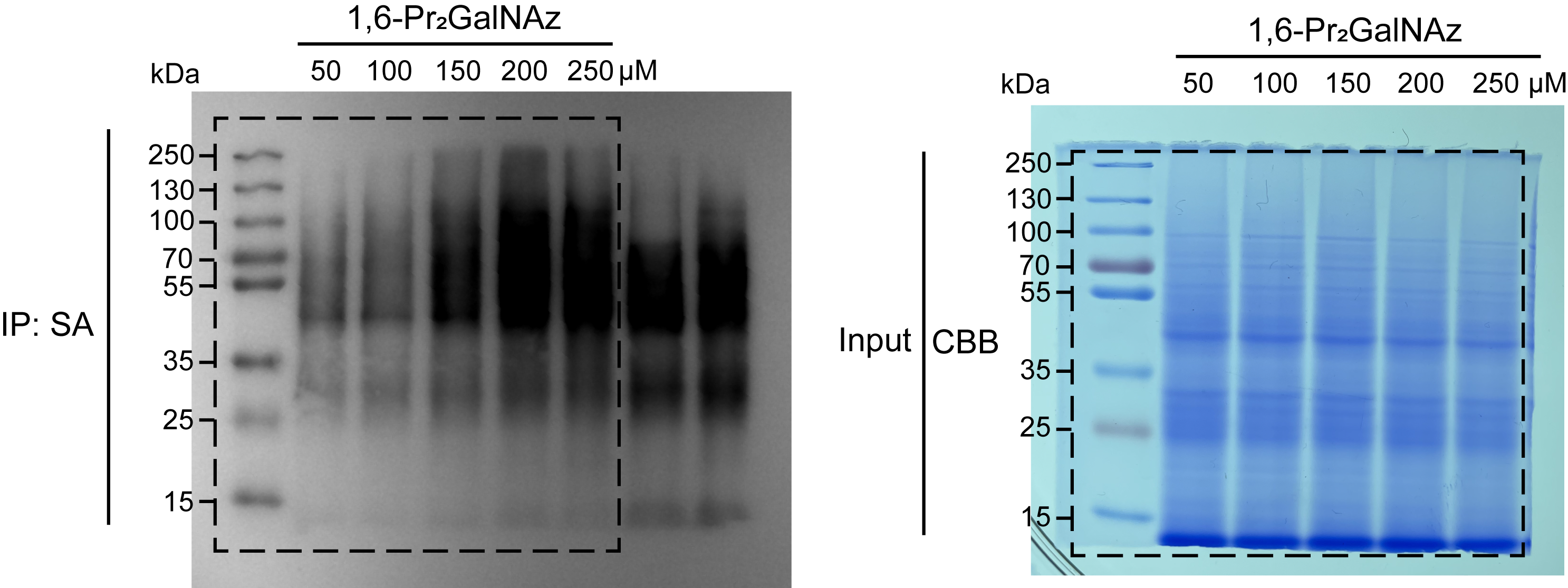
**

**Figure 1D:**

**
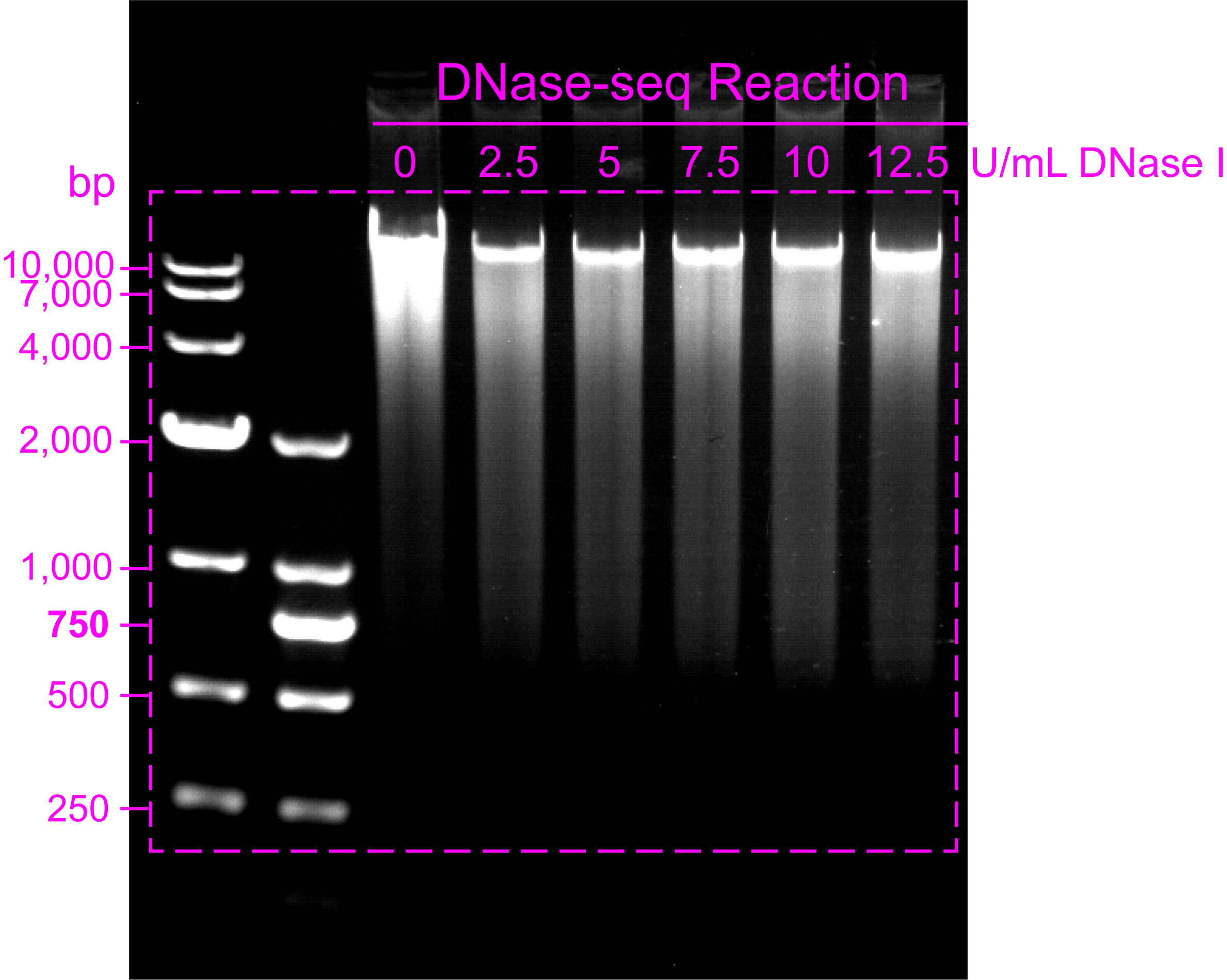
**

**Figure 1E:**

**
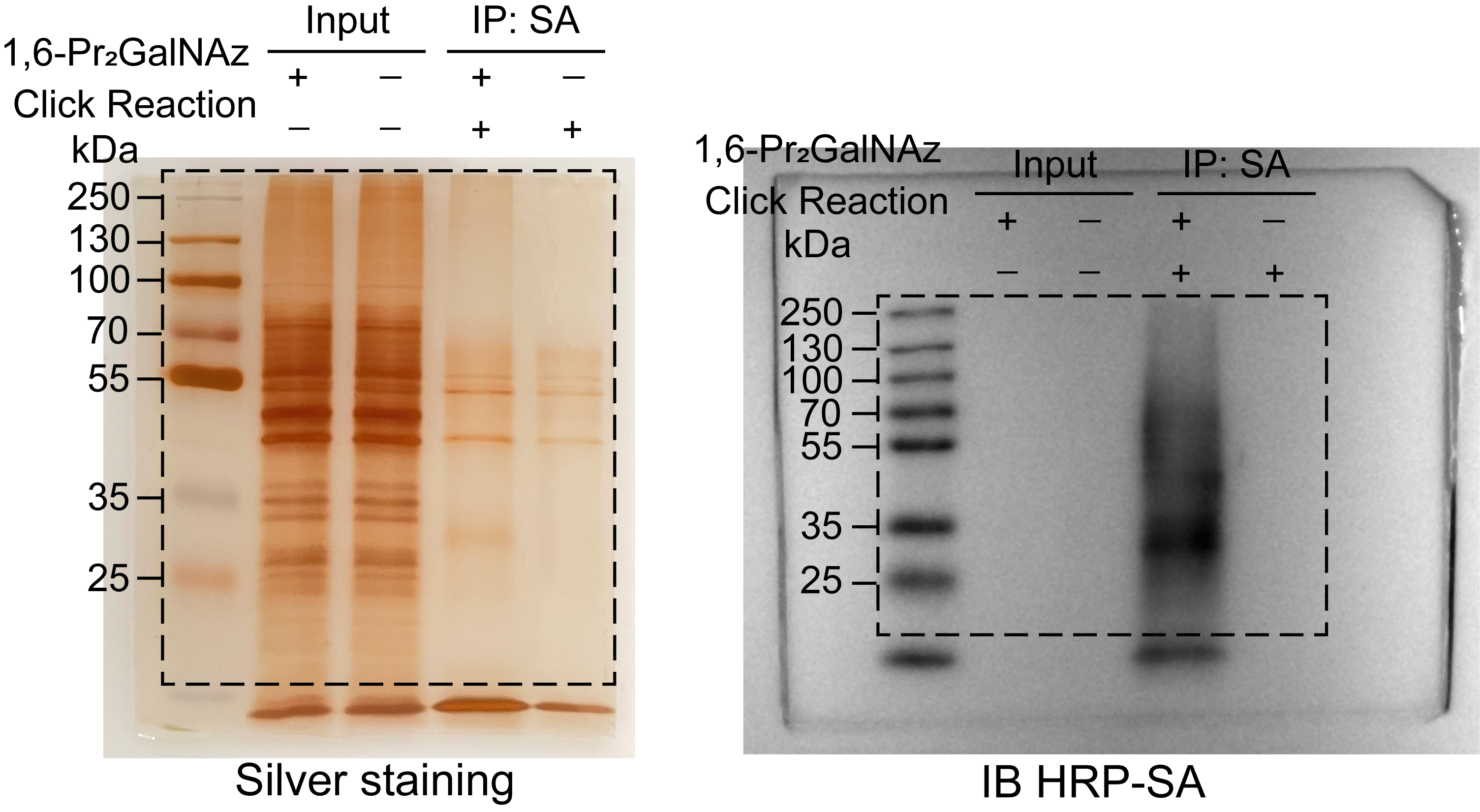
**
