# Supplementary material for: COCA-seq: genome-wide mapping of O-GlcNAc-associated open chromatin: COCA-seq maps O-GlcNAcylated open chromatin
Source: Acta Biochim Biophys Sin (Shanghai). 2025 Nov 24;58(5):975–88. doi: 10.3724/abbs.2025207 (PMC13214506; doi:10.3724/abbs.2025207)
Supplement: Supplementary_information_figures_and_tables.docx [file Supplementary_information_figures_and_tables.docx]

***Supplementary information***

This file includes:

**Supplementary Figure. S1-S3**.

**Supplementary Table. S1-S3**.


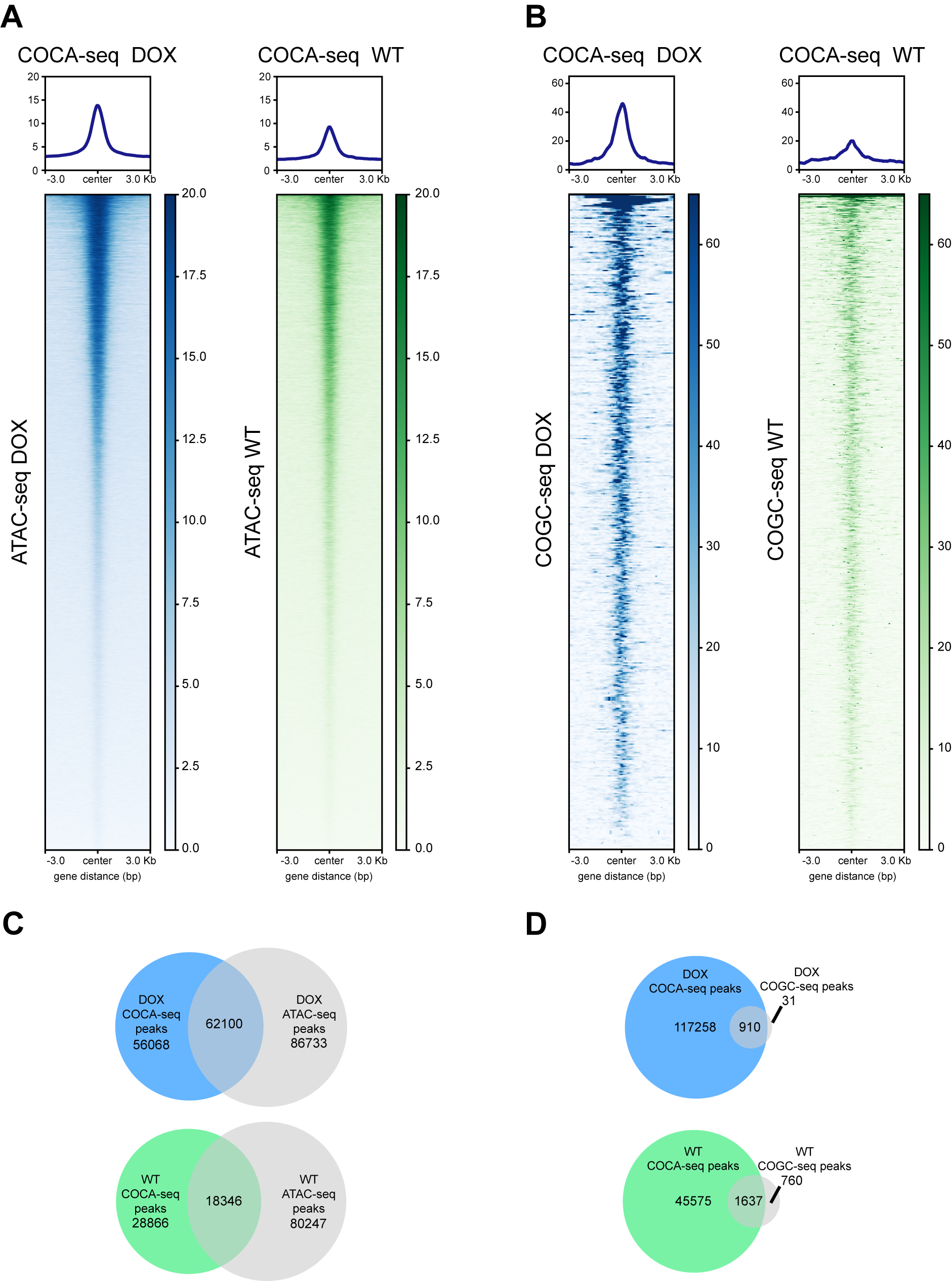


**Supplementary Figure S1. Validation of COCA-seq biological replicates in capturing O-GlcNAc-associated chromatin** (A-B) Independent biological replicates showing concordant patterns of read density profiles (top) and heatmap distributions (bottom) at genomic regions defined by ATAC-seq (A) and COGC-seq (B). The blue/green color indicates a high signal. (C-D) Overlap analysis confirmed similar intersection rates between COCA-seq peaks and chromatin regions identified by ATAC-seq (C) or COGC-seq (D) across replicates. All analyses were performed using identical bioinformatics pipelines as in Figure 2.


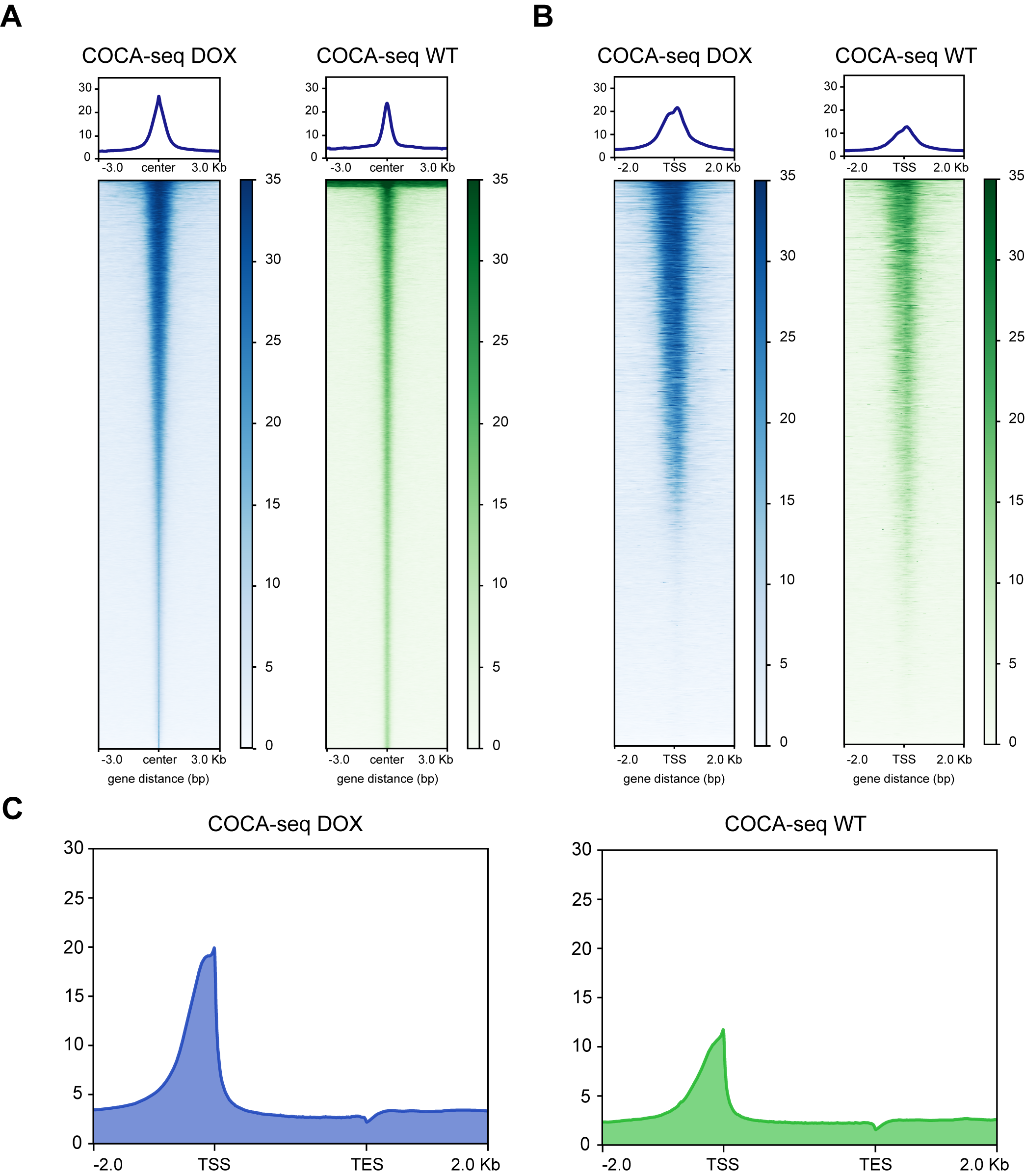


**Supplementary Figure S2. Consistent enrichment of COCA-seq signals at promoter regions in doxorubicin-resistant breast cancer cells** (A-B) Independent biological replicates analysis of COCA-seq read density distribution: (A) Heatmaps and average profiles (±3.0 kb) centered at COCA-seq peak summits, recapitulating promoter-focused enrichment. (B) Uniform signal distribution (±2.0 kb) flanking TSSs, with blue/green color scales indicating equivalent intensity metrics as Figure 3. (C) Reproducible genome-wide COCA-seq profiles spanning from TSS to TES regions (hg19). All analyses employed the same bioinformatics pipelines as in Figure 3.


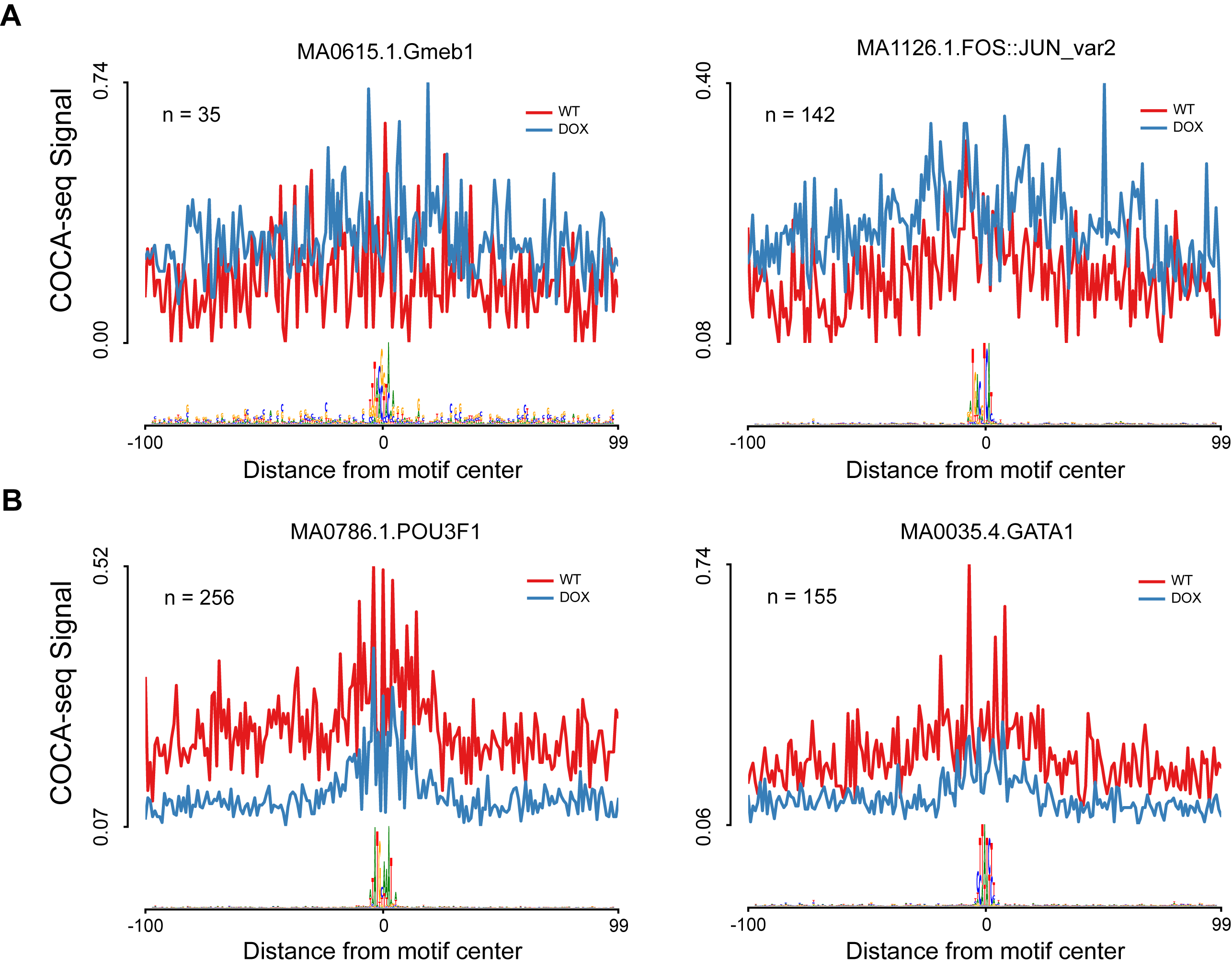


**Supplementary Figure S3. Additional TF binding motifs enriched in COCA-seq peak regions** (A-B) COCA-seq signal-associated motif analysis for more differential TFs: (A) Gmeb1 and FOS::JUN_var2 binding motifs in DOX. (B) POU3F1 and GATA1 motifs in WT. Greater COCA-seq signal intensity corresponds to higher predicted TF activity.

**Supplementary Table S1. List of used RT-qPCR primers**

| Gene | Forward | Reverse |
| --- | --- | --- |
| MFAP3L | GCAACTCTCAGTCTCTCTGCTCC | ACCCACTTCTGCAGGTCCGA |
| CTPS1 | AATACACGAAGTTCTCAGACTCCTATG | GCTCCAAGTCCGCAGAATCTATG |
| CSE1L | GGAACTGGAGAATTGTTGAAGATGAAC | TGAATTTGCTCTGGGCTGCTAAG |
| GAPDH | ACCCACTCCTCCACCTTTGAC | TGTTGCTGTAGCCAAATTCGTT |

**Supplementary Table S2. List of used COCA-qPCR primers**

| Gene | Forward | Reverse |
| --- | --- | --- |
| MFAP3L | CTGTAGTGTTTATGGCATCCCTGAC | CTCCTCTCTCCTTCTCATCCTCTTC |
| CTPS1 | GCGTGAGTTCCCAAACAGCCTCC | GCGTGTCTGCCCCTCTGCTT |
| CSE1L | CCTCCTCCGTTTCTGTATCCCCACGAG | GGAGCGCCTCATCCCTTCCTCA |

**Supplementary Table S3. List of the relative pathways from KEGG with the highest enrichment degree of BPGs**

| DOX-BPGs | | |
| --- | --- | --- |
| Description | Enrichment | -Log_10_*p* |
| Relaxin signaling pathway | 1.565 | 7.546 |
| Ubiquitin mediated proteolysis | 1.499 | 6.562 |
| Lysosome | 1.476 | 5.697 |
| HIF-1 signaling pathway | 1.462 | 4.584 |
| Endocytosis | 1.455 | 9.420 |
| p53 signaling pathway | 1.451 | 3.183 |
| Cell cycle | 1.422 | 5.396 |
| Apoptosis | 1.416 | 4.641 |
| FoxO signaling pathway | 1.405 | 4.330 |
| ErbB signaling pathway | 1.402 | 2.982 |
| Aldosterone synthesis and secretion | 1.400 | 3.282 |
| mTOR signaling pathway | 1.377 | 4.464 |
| Estrogen signaling pathway | 1.344 | 3.453 |
| Autophagy-animal | 1.343 | 4.054 |
| Breast cancer | 1.342 | 3.606 |
| AMPK signaling pathway | 1.338 | 3.012 |
| Ras signaling pathway | 1.322 | 4.883 |
| MAPK signaling pathway | 1.309 | 5.588 |
| cAMP signaling pathway | 1.298 | 4.108 |
| PI3K-Akt signaling pathway | 1.274 | 5.397 |
| WT-BPGs | | |
| Description | Enrichment | -Log_10_*p* |
| Mucin type O-glycan biosynthesis | 2.057 | 3.801 |
| Inflammatory mediator regulation of TRP channels | 1.887 | 6.965 |
| Glycosaminoglycan biosynthesis-heparan sulfate/heparin | 1.763 | 1.695 |
| Other types of O-glycan biosynthesis | 1.725 | 2.660 |
| Arginine biosynthesis | 1.686 | 1.431 |
| Thyroid hormone synthesis | 1.645 | 3.261 |
| Adherens junction | 1.630 | 3.758 |
| Alanine, aspartate and glutamate metabolism | 1.620 | 1.772 |
| Folate transport and metabolism | 1.592 | 1.469 |
| Protein digestion and absorption | 1.578 | 3.636 |
| NF-kappa B signaling pathway | 1.578 | 3.636 |
| Tyrosine metabolism | 1.567 | 1.546 |
| Rap1 signaling pathway | 1.563 | 6.414 |
| cAMP signaling pathway | 1.560 | 6.730 |
| Phospholipase D signaling pathway | 1.538 | 4.362 |
| Hormone signaling | 1.481 | 5.079 |
| cGMP-PKG signaling pathway | 1.444 | 3.531 |
| Cell adhesion molecules | 1.432 | 3.285 |
| Cysteine and methionine metabolism | 1.424 | 1.385 |
| Regulation of actin cytoskeleton | 1.413 | 4.176 |
